# Predictors of Beta-Blocker Intolerance and Mortality in Patients After Acute Coronary Syndrome

**DOI:** 10.1371/journal.pone.0077747

**Published:** 2013-10-22

**Authors:** Laercio Martins De Stefano, Alex Lombardi Barbosa Ferraz, Ana Lúcia dos Anjos Ferreira, Ana Lúcia Gut, Ana Lúcia Cogni, Elaine Farah, Beatriz Bojikian Matsubara

**Affiliations:** Faculdade de Medicina de Botucatu, UNESP, Departamento de Clínica Médica, Distrito de Rubião Junior, São Paulo, Brazil; S.G.Battista Hospital, Italy

## Abstract

**Purpose:**

To investigate the predictors of intolerance to beta-blockers treatment and the 6-month mortality in hospitalized patients with acute coronary syndrome (ACS).

**Methods:**

This was a single-center, prospective, and longitudinal study including 370 consecutive ACS patients in Killip class I or II. BBs were prescribed according to international guidelines and withdrawn if intolerance occurred. The study was approved by the institutional ethics committee of our university. Statistics: the clinical parameters evaluated at admission, and the related intolerance to BBs and death at 6 months were analyzed using logistic regression (p<0.05)in PATIENTS.

**Results:**

BB intolerance was observed in 84 patients and was associated with no prior use of statins (OR: 2.16, 95%CI: 1.26–3.69, p= 0.005) and Killip class II (OR: 2.5, 95%CI: 1.30-4.75, p=0.004) in the model adjusted for age, sex, blood pressure, and renal function. There was no association with ST-segment alteration or left anterior descending coronary artery plaque. Intolerance to BB was associated with the greatest risk of death (OR: 4.5, 95%CI: 2.15–9.40, p<0.001).

**Conclusions:**

After ACS, intolerance to BBs in the first 48 h of admission was associated to non previous use of statin and Killip class II and had a high risk of death within 6 months.

## Introduction

 Beta-blocker (BB) treatment decreases the mortality rate in patients with acute coronary syndrome (ACS). Therefore, its use is currently recommended as a class I-A indication in clinical practice guidelines[1,2].

 Several strategies have been developed to encourage the prescription of BBs early after the diagnosis of ACS. However, it has been reported that nearly 22% of eligible patients do not receive the medication [3–5]. All patients with ACS are considered eligible regardless of the concomitant administration of fibrinolytics or primary angioplasty; being in Killip class I or II; and the absence of bradycardia (FC < 60 bpm), severe hypertension, and advanced atrioventricular block (AVB).

 However, the nonprescription of BBs might be due to intolerance to the medication rather than to nonadherence to evidence-based therapy [6]. The literature presents little information about the frequency that patients hospitalized with ACS and in Killip I or II classification do not receive BBs because of their intolerance to the drug, and how this effects mortality. Moreover, little is known about the clinical predictors of nontolerance to BBs during hospitalization for ACS.

 The present study aims to evaluate the characteristics associated with nontolerance to BBs in patients with ACS, and to identify its impact in the 6-month all-cause mortality.

## Methods

 The procedures were approved by The Research Ethics Committee of the Botucatu School of Medicine (FMB, UNESP; OF213/2004-CEP) and were conducted in accordance with the Declaration of Helsinki. All participants had ability to consent and signed an informed consent form. 

 This is a prospective and longitudinal observational study comprising 377 consecutive patients older than 18 years and admitted to the hospital with unstable angina (UA), non-ST-segment elevation myocardial infarction (NSTEMI), or ST-segment elevation myocardial infarction (STEMI) [7–12] diagnosed up to 48 h after the onset of symptoms. The patients were admitted to the intensive care unit of the Emergency Hospital and in the coronary unit of the Botucatu School of Medicine University Hospital from March 1, 2003 to December 31, 2006. Exclusion criteria were Killip class III or IV at admission, heart rate <60 bpm, systolic blood pressure (SBP) <100mmHg, and PR interval >0.24s, second or third AVB and history of asthma or severe obstructive pulmonary disease.

 After the ACS diagnosis, the patients underwent clinical and laboratory evaluations, according to the standardized protocols implemented in the intensive care unit at Botucatu Medical School for patients with ACS. All patients were administered with acetylsalicylic acid as well as submitted to mechanical or chemical reperfusion when indicated. Patients who fulfilled the inclusion criteria were treated with BBs. The medication used in all cases was metoprolol, following the international guidelines. Briefely, Intravenous metoprolol tartrate was given in 5 mg increments by slow intravenous administration (5 mg over one to two minutes), repeated every five minutes for a total initial dose of 15 mg (three doses). Patients who tolerate this regimen then received oral therapy beginning 15 min after the last intravenous dose (25 to 50 mg every six hours for 48 hours of metoprolol tartrate) followed by a maintenance dose of 100 mg twice daily. Patients who do not receive a beta blocker during the first 24 hours because of early contraindications were reevaluated for beta blocker candidacy for subsequent therapy. Oral metoprolol tartrate 25 to 50 mg every 6 to 12 hours, titrating upward as needed [8]. The doses were tittered up to the recommended full-dose. They also received angiotensin-converting enzyme inhibitors (ACEIs), unless contraindicated. The contraindications to ACEIs include arterial hypotension and severe renal dysfunction (serum creatinine level >2.5 mg/dL in men or >2.0 mg/dL in women). After the onset of treatment, patients were defined as nontolerant to BBs if they developed bradycardia, hypotension, AVB, or severe and symptomatic ventricular dysfunction to any dose of BB.

### Data collection

Gender, age, ST elevation, comorbidities (arterial hypertension, diabetes mellitus, dyslipidemia, overweight/obesity, renal failure), tobacco use, previous ACS, and pharmacological treatment at admission were recorded, as well as blood pressure, heart rate, Killip classification, and blood glucose level at admission. The culprit artery was determined with coronary angiography during the in-hospital period.

### Outcome measures

In the first analysis, the primary outcome was the intolerance to BB during the hospitalization period and the 6-month mortality thereafter.

### Statistical analysis

Data management and analysis were performed using SYSTAT 12.0 (SYSTAT Software Inc. 2007). Summary data are expressed as either mean (SD) or proportions. Univariate regression analysis was performed in order to assess which variables were independently associated with either BB treatment intolerance or 6-month all-cause mortality. Those variables that showed a statistically significant association with each outcome were introduced into a multivariate model. The analysis of BB intolerance included the variables age, gender, SBP, Killip class, serum creatinine, and use of ACEIs and statins. For the 6-month mortality analysis, age, history of ACS, intolerance to BBs, and the presence of atherosclerotic plaque in the left anterior descending (LAD) coronary artery were considered. The level of significance was set at 5%.

## Results

 During the follow-up period, 7 patients lost contact with the service and were excluded from the analysis. Therefore, 370 patients (224 men and 146 women; p< 0.001) were consecutively included. The demographic and clinical characteristics of the studied population are presented in Table 1. Male patients were younger than female ones (59 ± 12 years and ± 12.5 years, respectively; p < 0.001). Acute events diagnosed at hospital admission were UA in 33.7% (n= 124), NSTEMI in 32.6% (n= 122), and STEMI in 33.7% (n= 124).

**Table 1:** Demographic and clinical variables of the studied population.

**Characteristics**	**Total**	**Male**	**Female**	**p**
n	370	224	146	<0.001
Age (years)	62±12.6	59±12	65±12.5	<0.001
Acute event				
UA/NSTEMI	246 (66.5%)	142 (63.4%)	104 (71.2%)	
STEMI	124 (33.5%)	82 (36.6%)	42 (28.8%)	0.236
Reperfusion	124 (34.3%)	84 (37.5%)	43 (29.5%)	0.132
CV risk factors				
AH	263 (71%)	143 (63.8%)	120 (82.2%)	<0.001
Diabetes mellitus	122 (33%)	58 (25.8%)	64 (43.8%)	<0.001
Smoking	208 (56.2%)	147 (65.6%)	61 (41.8%)	<0.001
Dyslipidemia	165 (44.6%)	85 (37.9%)	80 (54.8%)	0.001
Obesity	134 (36.2%)	73 (32.6%)	61 (41.8%)	0.022
Previous event	130 (35.1%)	79 (35.3%)	51 (34.9%)	0.947
Ischemic wall				
Anterior	188	114	74	0.907
Inferior	108	70	38	0.324
Killip II	72 (19.5%)	40 (17.8%)	32 (21.9%)	0.002
SBP (mmHg)	130±23.5	130±25	130±25	0.930
Necrosis markers	241 (65.1%)	152 (67.8%)	90 (61.6%)	0.177
Glucose (g/dL)	124±55.6	120±54	131±58	0.06
Creatinine (g/dL)	1.30±1.12	1.39±1.34	1.14±0.76	0.024
Drug at admission				
Beta-blocker	271 (73.2%)	172 (76.8%)	99 (67.8%)	0.042
ACEI	288 (78.6%)	176 (78.6%)	112 (76.7%)	0.632
Statin	208 (56.2%)	122 (54.5%)	86 (58.9%)	0.398

UA: unstable angina; NSTEMI: non-ST-segment elevation myocardial infarction; STEMI: ST-segment elevation myocardial infarction; CV: cardiovascular; AH: arterial hypertension; SBP: systolic blood pressure; ACEI: angiotensin-converting enzyme inhibitor.

 Statistically significant differences were found between male and female patients in hypertension (p< 0.001), diabetes mellitus (p 0.001), smoking (p< 0.001), and dyslipidemia (p< 0.001). At the initial evaluation, 85.7% of men (n= 192) were in Killip I class, and 14.3% received the classification Killip II. Among the females, 72.6% (n= 160) were in Killip I and 27.4% (n= 40) were in Killip II (p= 0.002). Plasma creatinine was higher among males (1.39 ± 1.34 g/dL vs. 1.14 ± 0.76 g/dL; p= 0.024). Of the whole population, 76% received BBs (48% males [n= 172] and 28% females [n= 99]). Among the female patients, 30% did not tolerate any dose of BBs, whereas only 20% among the males showed intolerance (p = 0.042).

 Coronary angiography was performed in 348 patients. An atherosclerotic plaque causing an obstruction >60% in the LAD coronary artery, right coronary artery, and/or circumflex coronary artery was observed in 243 (69.8%), 180 (51.7%), and 160 (46%) patients, respectively. 

 The univariate logistic regression for the outcome “BB intolerance” indicated that the Killip II classification more than doubled the risk for non-tolerance to this therapy as compared to Killip I class. In addition, for each unit increase in plasma creatinine, the risk of BB intolerance increased by 30%. The nonuse of ACEIs and statins increased the risk of intolerance to BB use (Table 2). 

**Table 2:** Univariate regression analysis for the primary outcome “beta-blocker intolerance”.

**Variable**	**OR**	**CI_95%_**	**p**
Age (years)	1.024	(1.005–1.044)	0.016
Gender	0.603	(0.369–0.984)	0.043
Weight	0.991	(0.973–1.010)	0.361
ST elevation	0.825	(0.498–1.368)	0.456
AH	1.380	(0.819–2.324)	0.226
LV anterior wall_no	1.034	(0.640–1.671)	0.891
Diabetes mellitus	0.979	(0.583–1.644)	0.937
Smoking	1.217	(0.748–1.981)	0.428
Dyslipidemia	1.189	(0.728–1.942)	0.489
Obesity	1.037	(0.607–1.772)	0.895
Previous ACS	1.211	(0.724–2.026)	0.465
SBP	0.988	(0.978–0.999)	0.031
DBP	0.980	(0.964–0.997)	0.023
Killip_I	0.391	(0.224–0.681)	0.001
Glucose	1.003	(0.998–1.008)	0.207
Creatinine	1.296	(1.069–1.571)	0.008
ACEI_no	2.938	(1.680–5.139)	<0.001
Statin_no	2.265	(1.383–3.708)	0.001
ACS/DA disease	1.060	(0.615–1.824)	0.836

OR: odds ratio; CI_95%_: 95% confidence interval; AH: arterial hypertension; SBP: systolic blood pressure; DBP: diastolic blood pressure; ACEI: angiotensin converting enzyme inhibitor; ACS/DA disease: acute coronary syndrome with lesion in the anterior descending coronary artery.

  The results of the multivariate logistic regression analysis for the outcome “BB intolerance” including age, gender, systolic blood pressure serum, creatinine, non-use of ACEI, and statin are presented in Table 3. Killip class II remained associated with intolerance to BBs. The effects of the nonuse of ACEIs and statins persisted, as in the univariate regression analysis.

**Table 3:** Multivariate regression analysis for the primary outcome “beta-blocker intolerance”.

**Variable**	**OR**	**CI_95%_**	**p**
Age (years)	1.016	(0.993–1.039)	0.167
Gender	0.704	(0.402–1.233)	0.220
SBP	0.984	(0.973–0.996)	0.007
Killip II	2.530	(1.350–4.750)	0.004
Creatinine	1.228	(0.995–1.517)	0.058
ACEI_no	2.364	(1.262–4.428)	0.007
Statin_no	2.159	(1.263–3.693)	0.005

OR: odds ratio; CI_95%_: 95% confidence interval; SBP: systolic blood pressure; DBP: diastolic blood pressure; ACEI: angiotensin converting enzyme inhibitor.

 Death at 6 months was associated with age, medical history of arterial hypertension, Killip II at admission, increased serum creatinine, and the presence of a >60% lesion in the LAD coronary artery. In addition, in-hospital intolerance to BB increased the risk of death during follow up by 4.5 times (Table 4).

**Table 4:** Univariate regression analysis for death at6 months.

**Variables**	**OR**	**CI_95%_**	**p**
Age (years)	1.07	(1.037–1.104)	<0.001
Gender	0.58	(0.29 - 1.17)	0.126
Weight	0.98	(0.95–1.01)	0.169
ST elevation	0.68	(0.44–1.05)	0.082
AH	3.46	(1.19–10.04)	0.023
Diabetes mellitus	1.40	(0.68–2.85)	0.361
Smoking	1.04	(0.51–2.09)	0.923
Dyslipidemia	1.35	(0.67–2.70)	0.402
Obesity	1.22	(0.58–2.58)	0.593
Previous ACS	1.43	(0.70–2.89)	0.323
SBP	1.01	(0.99–1.02)	0.331
DBP	1.01	(0.99–1.04)	0.221
Killip>I	2.75	(1.31–5.78)	0.007
Necrosis marker	0.55	(0.27–1.11)	0.097
LV anterior wall	0.484	(0.229–1.025)	0.058
Glucose	1.00	(1.00–1.01)	0.663
Creatinine	1.353	(1.112–1.647)	0.003
Beta-blocker_no	4.500	(2.157–9.390)	<0.001
ACEI_no	2.284	(1.050–4.971)	0.037
Statin_no	1.759	(0.856–3.614)	0.124
ACS/LAD disease	4.380	(1.300–14.760)	0.017

OR: odds ratio; CI_95%_: 95% confidence interval; AH: arterial hypertension; SBP: systolic blood pressure; DBP: diastolic blood pressure; LV: left ventricular; ACEI: converting enzyme inhibitor; ACS/LAD disease: acute coronary syndrome with lesion in the left anterior descending coronary artery.

  Multivariate logistic regression indicated that female gender, medical history of arterial hypertension, Killip II class, increased serum creatinine, atherosclerotic plaque in the LAD coronary artery, and nonuse of BBs and/or ACEIs were independently associated with death at 6 months.

In the adjusted model including clinical variables and angiographic findings, we have found that intolerance to BBs and anterior descending coronary artery disease were the most important predictors of all-cause mortality (Table 4).

## Discussion

 The present study aimed to identify variables associated with intolerance to BB use in hospitalized ACS patients. We here show that some clinical parameters easily obtained in the emergency department on admission can predict such intolerance. In addition, intolerance to treatment with BB increases the risk of death at 6 months in these patients.

 Our study found that 23% of the ACS patients eligible to did not tolerate BB treatment. This result is similar to those described by other researchers worldwide [3–5,13]. However, the other studies suggested that patients who were not prescribed such drugs were in fact potential candidates for the treatment. For example, a Korean study has recently reported that the use of statins was limited to the equivalent amount of such medications that can be reimbursed from the local health system; in other words, in addition to the lack of prescriptions, the use of these drugs seems to be limited by economic factors [6]. 

 The results of the present study suggest that the nonprescription of the drugs is due to the intolerance to their use rather than to the nonobservance of guidelines. The patients who did not tolerate BB use were those in Killip II class, with intolerance to ACEI, and were not under statin treatment.

 The univariate regression analysis showed that beyond the Killip II classification, impaired renal function is also a predictor for the nonuse of BBs. Several studies have analyzed the association between impaired renal function and the treatment of ACS [14–17]. Most of those studies have demonstrated that patients with ACS who present with some degree of impaired renal function do not receive the optimal recommended therapeutic approach. There may be several causes for that beyond the hemodynamic conditions at the emergency department, such as the presence of comorbidities that can limit the use of appropriate drugs. In our population, we did not identify factors that would explain the intolerance to the recommended medication for ACS in patients with impaired renal function.

 The nonuse of ACEIs and statins was associated with BB intolerance. Concerning ACEI, arterial hypotension would explain this association. However, the association of the lack of statin prescription with the nonuse of BB was intriguing. Patients who were not treated with statins had double the risk of nontolerance to BB. As our results do not allow us to explain such association, we discuss some considerations about the subject as follows. 

 The use of statins is recommended for patients with ACS and also as an adjuvant drug in the primary prevention of the disorder [18–21]. The beneficial effects of statins for patients with stable or unstable ACS have been attributed to their rheological effects and antithrombotic activity while stabilizing atherosclerotic plaques. Improvement of endothelial function, reduction of circulating C-reactive protein, and reduction of thrombogenicity have also been reported as beneficial effects of statin use. Furthermore, some reports have suggested an enhanced benefit of statins when used in association with BBs, mainly before a cardiovascular or noncardiovascular surgery [22,23].

A gender-related difference was noticed in the treatment. This is in agreement with the reports of others stating that elderly female patients receive less aggressive therapies when hospitalized with ACS, maybe because aggressive prescriptions are discouraged in this specific patient group [24–26].

 It is known that immediate reperfusion therapy and the introduction of adjuvant medications such as BBs, ACEIs, and statins are highly relevant for the better management of patients with ACS [27]. In addition, the more aggressive use of BBs, ACEIs, and statins for secondary prevention contributes to the reduction of morbidity and mortality in ACS patients [10,28,29]. 

ACS patients presenting an unstable hemodynamic condition at admission or a history of previous ACS events, kidney failure, or overt heart failure received less adjuvant pharmacological approach, in accordance with other reports [30–32]. However, our results suggest that the lack of prescription of the optimal treatment is due to the patient’s medical condition rather than to the nonobservation of the guidelines [33,34]. 

 The overall 6-month mortality was associated with age, arterial hypertension, Killip II, elevated serum creatinine, atherosclerotic plaque in the LAD coronary, and the nonuse of BB. Notably, the lack of BB use during hospitalization more than quadrupled the risk of death, regardless of the ACS type.

 Altogether, the results of the present study suggest that the parameters associated with BB intolerance during hospitalization should be aggressively managed in order to allow the use of the drug. Therefore, we emphasize the need for an individualized therapeutic strategy that would allow enough hemodynamic stability to make BB prescription possible in ineligible patients.
